# Toxic Epidermal Necrolysis A Diagnostic Dilemma in Puerperium: A Case Report

**DOI:** 10.31729/jnma.4638

**Published:** 2019-10-31

**Authors:** Bidya Mahto, Meena Thapa, Saraswati Padhye

**Affiliations:** 1 Department of Obstetrics and Gynecology, Kathmandu Medical College and Teaching Hospital, Sinamangal, Kathmandu, Nepal.

**Keywords:** *diagnostic dilemma*, *puerperium*, *toxic epidermal necrolysis*

## Abstract

Toxic epidermal necrolysis is a potentially life threatening dermatologic disorder characterized by widespread erythema, necrosis and bullas, detachment of epidermis and mucous membrane resulting in exfoliation, possible sepsis and even death. The incidence of toxic epidermal necrolysis is 0.4-1.2 per million people. This is the first case report in Nepal of toxic epidermal necrolysis in puerperium. We present a case of a 28-years-old, P_1_L_1_ on fourth postoperative day following emergency lower segment caesarean section for cephalopelvic disproportion in latent phase of labour with uneventful antenatal period. She developed fever followed by rashes all over the body with hypotension, tachypnea and shortness of breath. Initially, she was diagnosed as a case of septic shock and transferred to intensive care unit from postnatal ward. She was managed with broad spectrum antibiotics and inotropes. But her condition further deteriorated. Her pustule fluid culture showed no growth. Later on, it was found to be the case of Toxic epidermal necrolysis and managed with vancomycin and corticosteroids under the supervision of gynecology, dermatology and medicine team. Her condition was improved and was discharged on her twenty-third postoperative day.

## INTRODUCTION

Toxic epidermal necrolysis (TEN) is a severe mucocutaneous reaction, usually to drugs, characterized by blistering and epithelial sloughing.^1^ It is rare, affecting approximately 1-2 cases per million per year and are considered medical emergencies as they are potentially fatal.^2^ They are characterized by mucocutaneous tenderness and typically hemorrhagic erosions, erythema and more or less severe epidermal detachment presenting as blisters and areas of denuded skin. The average reported mortality rate of TEN is 25-35%.^2^ There is an increased incidence in women, the female to male ratio being 2:1.^3^ It is the first case report in Nepal of Toxic Epidermal Necrolysis of 28 years, on 4^th^ postoperative day following emergency lower segment caesarean section (LSCS) with diagnostic dilemma.

## CASE REPORT

A 28 years old primigravida at 40 weeks 1 day of gestation was admitted with a diagnosis of false labour pain and labour induced the next day. She underwent emergency LSCS for non-progress of labour and delivered a live male baby weighing 4.3kg. She had received intravenous Cefazoline 1 gm first dose preoperatively and 3 doses thereafter 8 hourly, intravenous Metronidazole 2gm single dose postoperatively, prophylactically as per hospital protocol. Her intraoperative period was uneventful. She was perfectly alright till 2^nd^ postoperative day.

**Figure 1:**
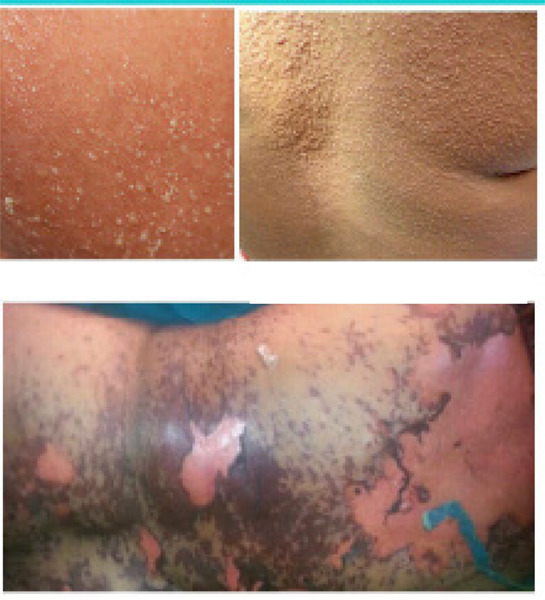
A,B,C. Initial pustular rash which later became generalized and fragile lesion of epidermis.

On 3^rd^ postoperative day she developed fever. Initially it was low grade later on it became high grade (up to 104°F). On her 4^th^ postoperative day, blood, urine, high vaginal swab culture tests were sent. Her total count was 20,600 with 91% neutrophil. Then she was given intravenous ceftriaxone 2gm once a day and an antipyretic. On 5^th^ postoperative day, despite of medications, she continued to have high grade fever. She developed pustular rashes on her neck which progressed to the trunk, abdomen, back and upper thigh with burning sensation(Figure 1A,B).

Dermatology and medicine consultation were done. Intravenous Ceftriaxone was stopped and Tab. Cefadroxil 500mg twice a day; Tab. Loratidine 10mg once at night; Fusidic acid 2% + Betamethasone Valerate 0.1% cream were started. On 6^th^ postoperative day she developed hypotension, tachycardia and tachypnea along with generalized pustular exanthematous rashes and high grade fever. Injection Hydrocortisone 100mg was added. All the above mentioned drugs were held. Same day at the evening, she suddenly developed shortness of breath with increasing tachycardia, hypotension and tachypnea. On auscultation, crepitations were heard over bilateral basal areas. She was shifted to ICU. Femoral venous line was inserted and was kept under continuous positive airway pressure (CPAP). Also ionotropes, subcutaneous Enoxaparin Sodium 40mg once a day and Intravenous clindamycin 600mg 8 hourly were started. On 7^th^ postoperative day, her total count raised to 30,400 along with deranged LFT reports. Dermatology consultation was done again. Provisional diagnosis of Toxic Epidermal Necrolysis with drug induced fever and lukemoid reaction was made. All antibiotics were stopped and intravenous Dexamethasone 8 mg once a day, Vancomycin 500 mg twice a day and Thiamine 200 mg twice a day were started.

On the next day patient improved dramatically and became afebrile. Skin rashes started to desquamate (Fig. 2) along with a decrease in burning sensation. On 9^th^ postoperative day all symptoms improved and ionotropes was stopped. Her pus culture, urine culture and blood culture reports were sterile. Tab. Prednisolone 40 mg once a day was started and injection vancomycin was continued. She was shifted to ward on her 13^th^ postoperative day. Later on, the patient developed wound infection and wound debridement and re-suturing were done. She recovered completely and was discharged on 25^th^ postoperative day.

## DISCUSSION

There have been many case reports on TEN. In Nepal, there are few case reports on TEN but no case reported for TEN in pregnancy. The cutaneous manifestations of TEN are usually preceded by non-specific symptoms such as fever, discomfort during swallowing, and stinging of eyes.^4,5^ Initially skin involvement include the pre-sternal, truncal region, face, palms and soles. In about 90% of patients, the involvement of the mucosa of the mouth, genital and/or gastrointestinal tract visible as erythema and erosions are present.^4,5^

Subsequently, lesion spread to involve the rest of the trunk and limbs; involvement of the palms and soles with target lesions is often prominent. Lesions increase in size and number over 5-7 days, tending to coalesce. Vesicles or fluid-filled blisters develop within lesioned skin.

Several drugs are at high risk of inducing TEN including: Allopurinol, Trimethoprim-Sulfamethoxazole and other Sulfonamide-antibiotics, Aminopenicillins, Cephalosporins, Carbamazepine, Phenytoin, Phenobarbital and NSAIDs.^2^

Diagnosis relies mainly on clinical signs together with the histological analysis of skin biopsy showing typical full-thickness epidermal necrolysis due to extensive keratinocyte apoptosis.

Due to high risk of mortality, management of patients with TEN requires rapid diagnosis, evaluation of the prognosis, identification and interruption of the culprit drug are necessary. Specialized supportive care, ideally in an intensive care unit with consideration of immunomodulating agents such as high-dose intravenous immunoglobin therapy 0.5-1 gm/kg daily for 3-4 days, systemic corticosteroid and Ciclosporin are required.^1^

Consent: JNMA Case Report Consent Form was signed by the patient and the original article is attached with the patient’s chart.

## Conflict of Interest:

None.
